# Editorial: The 30-year journey of the clinical hypertension

**DOI:** 10.1186/s40885-024-00286-5

**Published:** 2024-11-01

**Authors:** Jae-Hyeong Park

**Affiliations:** Department of Cardiology in Internal Medicine, School of Medicine, Chungnam National University, Chungnam National University Hospital, 282 Munhwa-ro, Jung-gu, Daejeon, 35015 Korea

## Introduction

The official scientific journal represents one of the most significant activities of the academic society. Its principal objective is to facilitate the advancement of academic knowledge. The journal functions as a conduit for researchers to disseminate and discuss their research findings, which is crucial for the advancement of knowledge within the field. Furthermore, journals facilitate academic exchange, whereby researchers publish their findings in journals for the benefit of other researchers, who can then read, discuss, and exchange opinions, thereby driving academic progress. Furthermore, journals contribute to the prestige and influence of a society, as the publication of high-quality papers raises the status of the society in its field. Finally, journals can help preserve and disseminate knowledge.

Consequently, the journal can be regarded as having a symbiotic relationship with the development of the society. In commemoration of the 30th anniversary of the Korean Society of Hypertension (KSH), we will provide a concise historical overview of the Clinical Hypertension.

## History of the clinical hypertension

### The clinical hypertension was born

The history of the Clinical Hypertension begins with the establishment of the KSH. The KSH was first established in July 1990 as the “Hypertension Research Society” within the Korean Society of Cardiology. Following the meeting of the promoters of the KSH in Seoul on March 17, 1994, Prof. Hark Joong Lee was appointed as president, Prof. Won Sang Yoo as vice president, Prof. Jung Sam Seo and Sam Soo Kim as advisors, and Prof. Chung Kyun Lee as secretary general, the articles of association of the KSH were drafted and established. The inaugural board meeting was held at the Scandinavian Club of the National Medical Center on Wednesday, April 27, 1994. At that time, Professor Young-Seol Kim of Kyung Hee University was appointed as the first editor-in-chief, and the journal was prepared for publication.

**Fig. 1:**
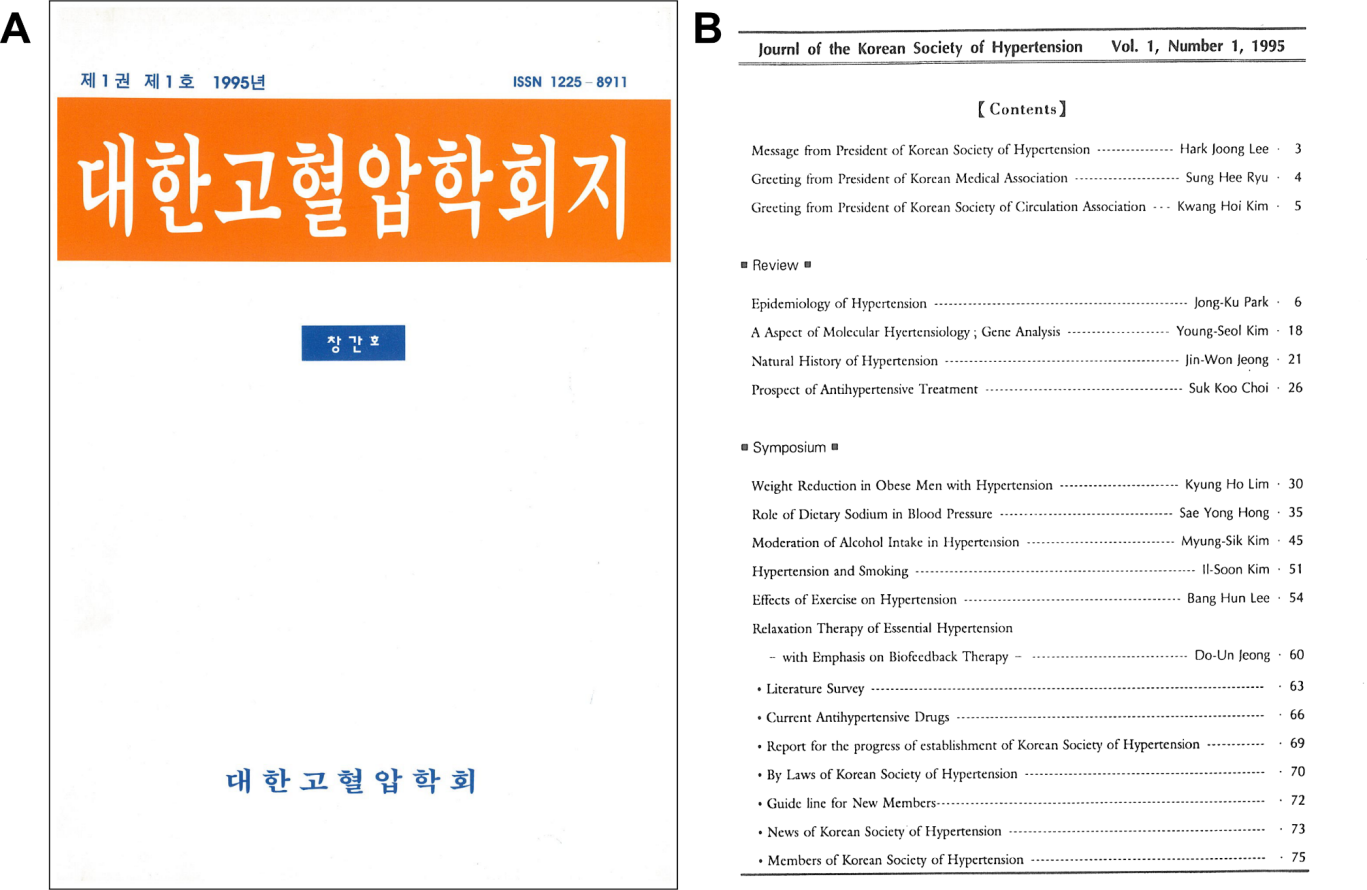
Cover (**A**) and table of contents (**B**) of the first issue of the Journal of the Korean Society of Hypertension

The inaugural issue of the Journal of the Korean Society of Hypertension (pISSN 1225–8911) was published in 1995 under the title “대한고혈압학회지 (Korean, The Journal of the Korean Society of Hypertension)” (Fig. 1).

One special issue and three original papers were published in the first issue, and the name ‘대한고혈압학회지 (Korean, The Journal of the Korean Society of Hypertension’ was used until 2003. The editor-in-chief, professor Young-Seol Kim, served until 1998, and in 1998, the name was changed from editor-in-chief to publication director at the sixth board meeting of KSH. The second editor-in-chief of the Journal of the Korean Hypertension Society was professor Suk-Koo Choi of Inje University, who served until 2001. Professor Jae Woo Lee of Koshin University, who was appointed as the ninth director of publications of KSH, became the third editor-in-chief of the Journal of the Korean Society of Hypertension in 2002, and he changed the official name of the journal to the ‘Journal of the Korean Society of Hypertension (English)’ in 2004.

### The journal grew into a full-fledged academic journal

The growth period of the Journal of the Korean Society of Hypertension began in 2004, when the official name was changed to ‘Journal of the Korean Society of Hypertension (English)’ and professor Kyu-Hyung Ryu of Konkuk University, who was appointed as the 12th director of publication in 2005, became as the fourth editor-in-chief. Previously, the term of the editor-in-chief of the journal ranged from two to four years, but with the fourth editor-in-chief, the term was changed to more than five years. During his eight-year tenure from 2005 to 2012, professor Yoo set the framework for the journal and improved the quality of articles. In 2011, he changed the official name of the journal to the Journal of the Korean Society of Hypertension and made serious efforts to English-language the journal, which was listed on Koremed, a domestic article search engine, in February 2011 and was listed as a candidate journal for the National Research Foundation (NRF) of Korea in February 2012.

### With the English-language edition, the Journal embarked on a path of full-scale globalization

Following the appointment of professor Jong-Won Ha of Yonsei University as the fifth editor-in-chief in 2013, the Journal of the Korean Society of Hypertension commenced a comprehensive process of globalization. In September 2014, he renamed the journal from “Journal of the Korean Society of Hypertension” to “Clinical Hypertension,” changed the publisher from a domestic publisher to a foreign publisher (Springer), and changed the publication format to the internet in 2015. As a consequence of these modifications, the journal was declined as a potential candidate for the NRF of Korea in July 2015, which recognizes journals that publish exclusively in print. However, it was subsequently included in PubMed, a global article search engine, in May 2016.

### Through systematic qualitative growth, the journal has been listed in Scopus and ESCI

Professor Wook-Jin Chung of Gacheon Medical University served as the sixth Editor-in-Chief for a period of five and a half years, from 2017 onwards. During this tenure, he hired an editorial staff, established and regularly held editorial board workshops and editorial sessions at conferences, interacted with and received assistance from experts in both domestic and international journals, and worked to systematically improve the quality of the KSH’s journal, Clinical Hypertension. Consequently, the journal was registered as candidate for journals of NRF of Korea in October 2019 (it was subsequently included in scholarly journal of NRF of Korea, currently), and it was also listed in the ESCI by Clarivate in May 2021 and in the SCOPUS by Elsevier in October 2021. Moreover, Editor-in-Chief Wook-Jin Chung endeavored to preserve the journal’s historical record by making articles published prior to 2015, when online publication commenced, accessible on the KSH website.

## Current status of the clinical hypertension

**Fig. 2:**
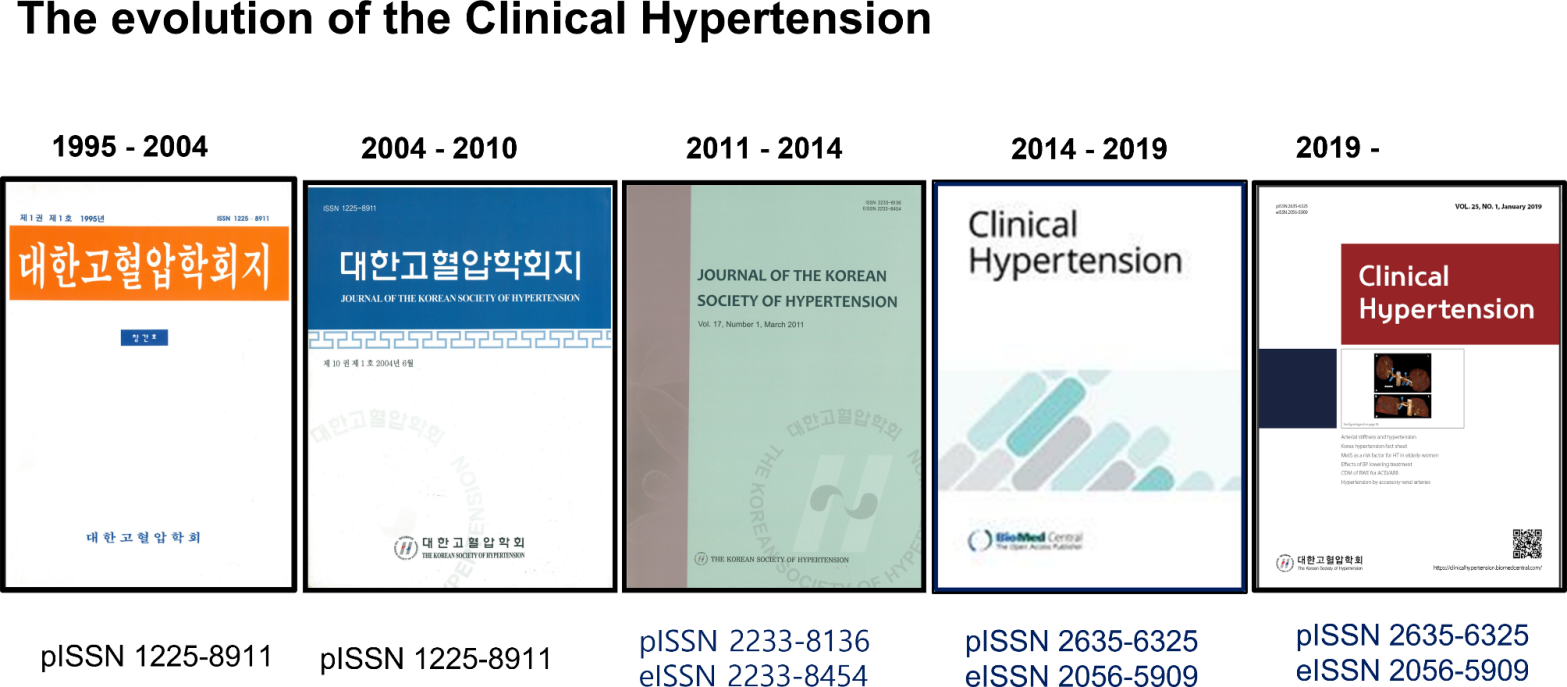
The evolution of clinical hypertension

The official journal of the KSH is Clinical Hypertension, which uses the editorial system of SpringerNature, a subsidiary of BMC, and is an open access journal that is free and conveniently available to all. Since 2023, professor Wook-Bum Pyun of Ewha Womans University has been serving as the seventh editor-in-chief (Fig. 2).

**Fig. 3:**
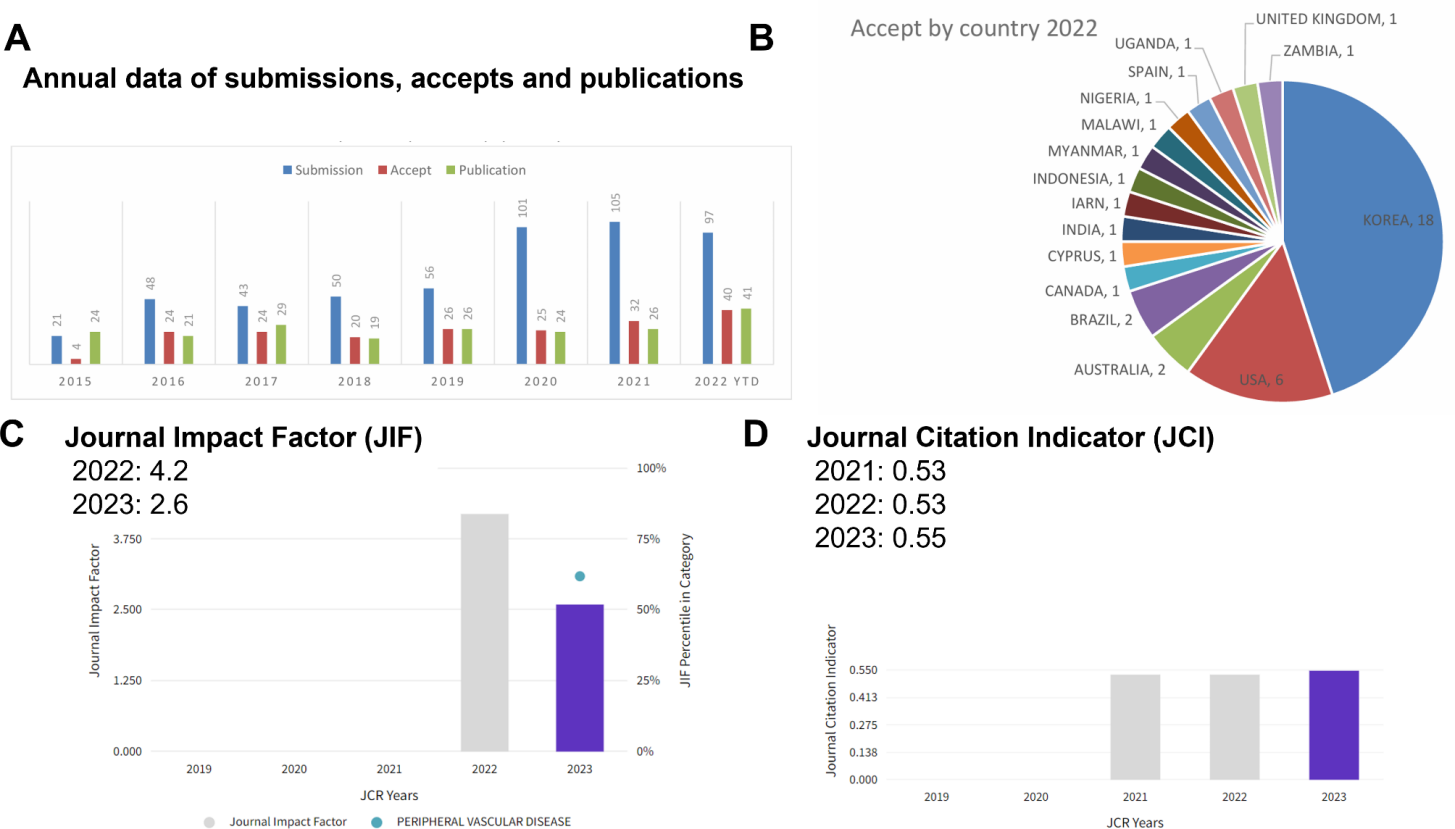
Current status of clinical hypertension. (**A**) Annual data of submissions, accepted articles and publications. (**B**) Countries of accepted articles. (**C**) Trend of Journal Impact Factor (JIF). (**D**) Trend of Journal Citation Indicator (JCI)

As of 2023, more than 150 manuscripts were submitted, and 33 articles submitted from more than 9 major countries were published (Fig. 3A, and B). As of 2023, approximately 400,408 articles were downloaded from the Internet, and the journal’s impact factor (JCI, Clarivate) as of 2023 was 2.6, ranking 37th (Q2) among 96 journals in the field of peripheral vascular disease worldwide, and the JCR was 0.55, ranking 45th (Q2) among 96 journals in the field of peripheral vascular disease worldwide (Fig. 3C, and D). The CiteScore based on Scopus is 5.4, ranking 100th out of 387 cardiology/cardiovascular journals in medicine and 52nd out of 167 journals in internal medicine.

## Conclusions

**Fig. 4:**
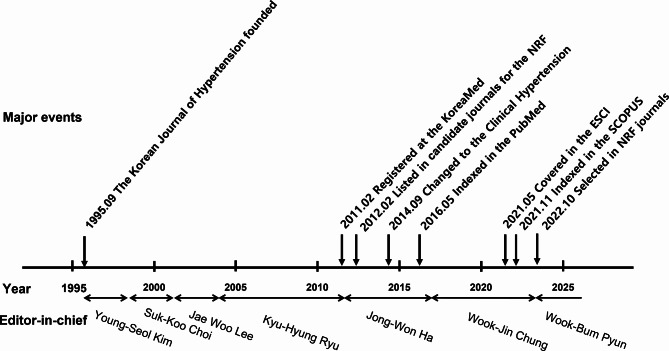
Major events and editors-in-chief by year. NRF: National Research Foundation

The Journal of the Korean Society of Hypertension was founded in 1995 and has a 30-year history, and over the years, with the full support of the KSH, the dedication of the six editors-in-chief, and the unremitting efforts of the editorial board members and members of the KSH, it has become one of the world’s leading journals through Pubmed, Scopus, ESCI, and the NRF of Korea (Fig. 4).

The current editor-in-chief, professor Wook-Bum Pyun, is trying to improve the quality of the journal by promoting it to the SCIE and listing it in the Medline. The status of the journal is related to the status of the society, so it is necessary for the society to fully support the journal along with the development of the journal. We look forward to the future development of the KSH and the Clinical Hypertension.

## Data Availability

Not applicable.

## Declarations

### Competing of interests

Jae-Hyeong Park serves on the editorial boards as a deputy editor in the Clinical Hypertension.

